# Earlier-Phased Cancer Immunity Cycle Strongly Influences Cancer Immunity in Operable Never-Smoker Lung Adenocarcinoma

**DOI:** 10.1016/j.isci.2020.101386

**Published:** 2020-07-18

**Authors:** Hong Kwan Kim, Je-Gun Joung, Yoon-La Choi, Se-Hoon Lee, Byung Jo Park, Yong Soo Choi, Daeun Ryu, Jae-Yong Nam, Mi-Sook Lee, Woong-Yang Park, Soohyun Hwang, Hongui Cha, Hong Sook Kim, Sanghyuk Lee, Yeonjoo Jung, Jong Eun Lee, Junsang Doh, Soonmyung Paik, Jung Hee Kang, Jinseon Lee, Jhingook Kim

**Affiliations:** 1 Department of Thoracic and Cardiovascular Surgery, Samsung Medical Center, Sungkyunkwan University School of Medicine, 81 Irwon-ro, Gangnam-gu, Seoul 06351, Korea; 2 Samsung Genome Institute, Samsung Medical Center, Seoul, Korea; 3 Department of Pathology and Translational Genomics, Samsung Medical Center, Sungkyunkwan University School of Medicine, Seoul, Korea; 4 Division of Hematology-Oncology, Department of Medicine, Samsung Medical Center, Sungkyunkwan University School of Medicine, Seoul, Korea; 5 Department of Health Science and Technology, Samsung Advanced Institute of Health Science and Technology, Sungkyunkwan University School of Medicine, Seoul, Korea; 6 Laboratory of Cancer Genomics and Molecular Pathology, Samsung Biomedical Research Institute, Samsung Medical Center, Seoul, Korea; 7 Department of Life Science, Ewha Womans University, Seoul, Korea; 8 Ewha Research Center for Systems Biology, Ewha Womans University, Seoul, Korea; 9 DNA Link Inc., Seoul, Korea; 10 Department of Materials Science and Engineering, Seoul National University, Seoul, Korea; 11 Severance Biomedical Science Institute, Yonsei University College of Medicine, Seoul, Korea; 12 Samsung Biomedical Research Institute, Samsung Medical Center, Sungkyunkwan University School of Medicine, 81 Irwon-ro, Gangnam-gu, Seoul 06351, Korea

**Keywords:** Immunology, Cancer Systems Biology, Genomics

## Abstract

Exome and transcriptome analyses of clinically homogeneous early-stage never-smoker female patients with lung adenocarcinoma were performed to understand tumor-T cell interactions and immune escape points. Using our novel gene panels of eight functional categories in the cancer-immunity cycle, three distinct subgroups were identified in this immune checkpoint blockade-refractory cohort by defective gene expression in two major domains, i.e., type I interferon production/signaling pathway and antigen-presenting machinery. Our approach could play a critical role in understanding immune evasion mechanisms, developing a method for effective selection of rare immune checkpoint blockade responders, and finding new treatment strategies.

## Highlights

• GMM-based clustering of cancer-immune gene expression helps to find LUAD classifiers
• Classifiers are found in the earlier-phased cancer-immunity cycle
• Classifiers include ubiquitination, Ag-presenting machinery, and type I IFN signaling
• Tumor-intrinsic classifiers strongly influence the outcome of cancer immunity

## Graphical Abstract

**Figure:**
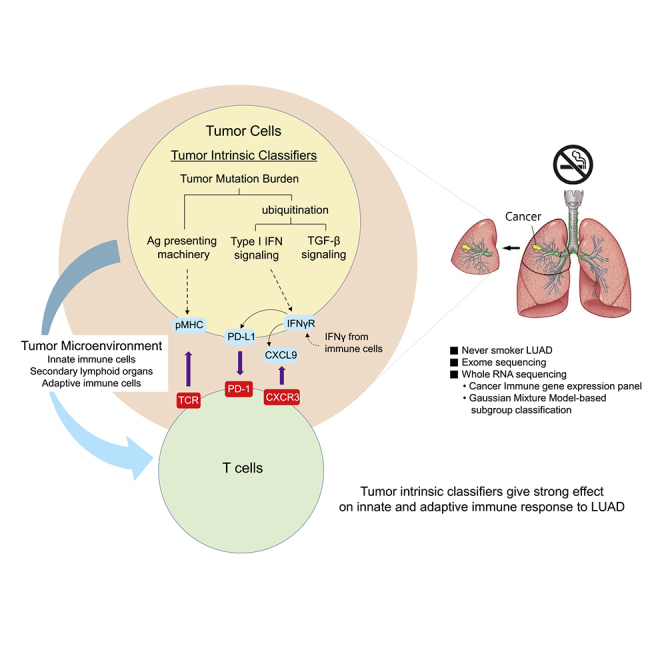

## Introduction

Antibody-based immunotherapies that modulate host immune responses against tumors have provided a paradigm shift in the treatment of non-small cell lung cancer (NSCLC) (Topalian et al., 2015). Specifically, immune checkpoint blockades (ICB) that target the programmed cell death protein 1 (PD-1), programmed cell death ligand-1 (PD-L1), or cytotoxic T-lymphocyte-associated protein 4 (CTLA-4) have led to dramatic clinical benefit with durable response (Reck et al., 2016; Rittmeyer et al., 2017; Schadendorf et al., 2015). However, the majority of patients do not respond to ICB (Sunshine and Taube, 2015), and thus there have been enormous efforts in identifying predictive biomarkers of treatment response (Carbone et al., 2017; Havel et al., 2019; McGranahan et al., 2016; Rizvi et al., 2015; Tumeh et al., 2014). Currently available predictive biomarkers include PD-L1 expression (Havel et al., 2019), tumor-infiltrating lymphocytes (Tumeh et al., 2014), and tumor mutational burden (TMB) (Carbone et al., 2017; McGranahan et al., 2016; Rizvi et al., 2015). In fact, a significant number of patients with PD-L1-positive results failed to respond to ICB (Rittmeyer et al., 2017). Furthermore, about 10% patients with PD-L1-negative results paradoxically responded to ICB (Rittmeyer et al., 2017; Sunshine and Taube, 2015).

In general, human tumors associated with environmental mutagens, such as smoking, are more likely to have a higher TMB (Govindan et al., 2012). Smoking may increase the mutational load, leading to neoantigen expression in tumor cells and helping to maintain an inflammatory environment, resulting in interferon gamma (IFNγ)-driven expression of PD-L1 (Taube et al., 2012). However, in parallel with smoking cessation (Pallis and Syrigos, 2013), the proportion of never smokers among patients recently diagnosed with NSCLC is continuously growing, currently exceeding 15% (Pelosof et al., 2017). Of NSCLC, lung adenocarcinoma (LUAD) more commonly affects never smokers than does squamous cell carcinoma (Pesch et al., 2012). Never-smoker patients with LUAD are characterized by a higher rate of strong driver genetic alterations, especially epidermal growth factor receptor (*EGFR*) mutation (Pallis and Syrigos, 2013). Constitutive activation of EGFR pathway could resultantly activate the downstream PI3K/AKT signaling pathway, upregulating PD-L1 expression (Akbay et al., 2013). Therefore, *EGFR*-mutant NSCLC is expected to be more responsive to PD-1 or PD-L1 ICB. However, most clinical trials consistently demonstrated no survival benefit from ICB in *EGFR*-mutant tumors, compared with conventional chemotherapy (Lee et al., 2017; Rittmeyer et al., 2017).

The success of anti-tumor immunotherapy requires a comprehensive understanding of the complex interactions within the cancer-immune system. To maintain immunological homeostasis, both innate and adaptive immune cells in the cancer-immunity cycle must work in a concerted manner (Chen and Mellman, 2017). Tumor growth and cancer metastasis could result from immune escape by any failure in this cycle, leading to T cell infiltration, tumor recognition, and killing. Therefore, identifying defective steps in the cancer-immunity cycle is the basis of both understanding the mechanism and developing possible approaches to overcome immune evasion. Thus, for precise assessment of this complex interaction within the cancer-immunity system, multi-omics profiling of changes at the DNA, RNA, and protein levels is required. To this end, exome and transcriptome analyses were conducted of surgically resected tumor specimens of never-smoker patients with LUAD to elucidate their underlying immune escape mechanism.

## Results

### Development of Novel Cancer-Immune Gene Panel

A novel cancer-immune gene panel was created from extensive literature review, which included the innate and adaptive host immune function with tumor microenvironment (TME), to identify significant immune escape points in this cohort. We were unsuccessful in clustering the whole-transcriptome profile of this cohort using several of the currently available immune cell signatures (Bindea et al., 2013; Danaher et al., 2017; Lyons et al., 2017; Sweis et al., 2016), which in fact could be regarded as an alternative to pathological immunophenotyping (Figure S1).

In general, tumor cells expressing a repertoire of immunogenic tumor-specific antigens (neoantigens) induce a prolific immune response. Therefore, tumors with low level of mutation burden, i.e., small repertoire of neoantigens, are very likely to have disadvantages in their immune responses, which can be further exacerbated by any failure in subsequent cancer-immune processes. Thus, we extensively reviewed major factors and processes playing an important role in anti-tumor immune function. Furthermore, we selected 546 immune function-related genes from the literature and classified them into the following eight functional categories in the cancer-immunity cycle: (1) ubiquitination/deubiquitination system, including E3 ubiquitin ligases playing a critical role in various signaling processes (Panel A); (2) type I IFN production/signaling pathway involved in the pro-inflammatory response (Panel B); (3) antigen-processing/presenting machinery, including proteasomal processing, expression of major histocompatibility complexes (MHCs) and their accessory molecules, and assembly of the antigenic peptide on MHC molecules (Panel C); (4) transforming growth factor (TGF)-β signaling pathway involved in anti-inflammatory immune-suppressive response (Panel D); (5) natural killer (NK) cell activation for enrichment of mature dendritic cells (DCs) that efficiently present neoantigens (Panel E); (6) activation of antigen-presenting cells by CD4 T cells in the lymph nodes (Panel F); (7) immune status of TME, in which tumor cells interact with immune cells and dynamic balance of pro- and anti-inflammatory signals takes place (Panel G); and (8) CD8 T cell activation, trafficking, infiltration, recognition, and killing of neoantigen-specific tumor cells (Panel H) (Figure S3 and Data S1).

### Three Distinctive Subgroups Based on Gene Panel Expression

**Figure 1:**
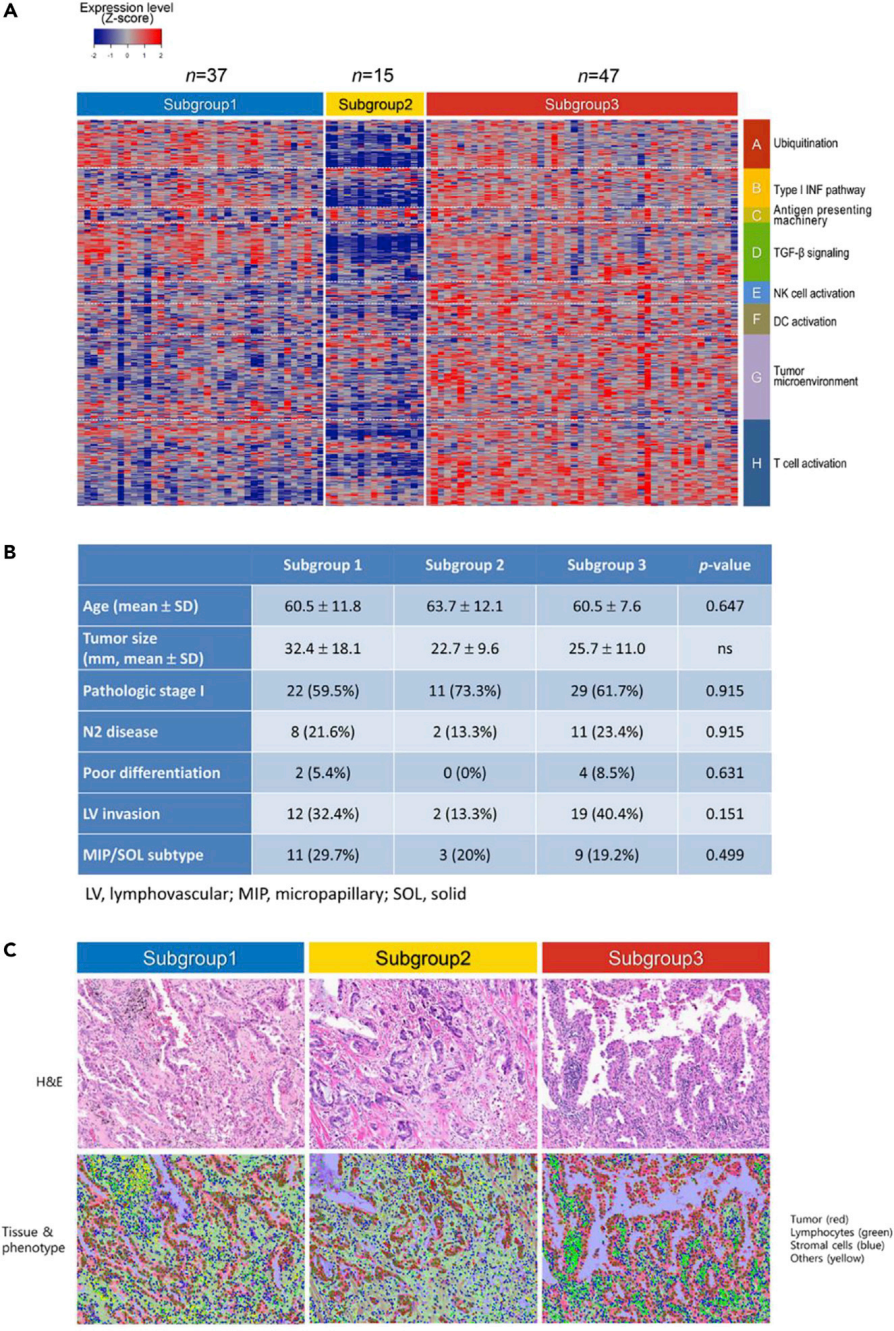
Three Subgroups of Never-Smoker Patients with LUAD with Clinicopathological Homogeneity (A) Three distinct subgroups of cancer-immune gene expression in never-smoker lung adenocarcinoma. Whole-transcriptome sequencing data from 99 never-smoker patients with lung adenocarcinoma were analyzed by Gaussian Mixture Model using eight different gene panels (546 in total) in the cancer-immunity cycle (Figure S3 and Data S1), revealing three subgroups. Subgroup 1 was defective in antigen-presenting machinery, NK cell activation, and type I IFN production (later named as subgroup with ubiquitination functioning, deregulated type I IFN production, and defective antigen presentation type). Subgroup 2 was defective in ubiquitinating enzymes expression, and the type I IFN and TGF-β signaling pathways were significantly downregulated (later named as subgroup with defective ubiquitination, type I IFN signaling, antigen presentation type). Subgroups 1 and 2 showed mutually exclusive gene expression patterns within panels of the cancer-immunity cycle. Subgroup 3 maintained relatively high levels of MHC class II and other cancer-immune gene expression (later named as subgroup with ubiquitination, type I IFN pathway, antigen presentation functioning) (Table 1 and Data S2). (B) Comparison of clinicopathological characteristics between subgroups. (C) Spatial distribution of lymphocytes in tumor/stromal area. H&E slide image showed very few lymphocytes in the tumor nest (first row), and most distributed in the stromal areas (green, second row) across subgroups within the representative region of interest. The patterns of tumor-infiltrating lymphocytes in each subgroup were indistinguishable by routine pathological readings.

We applied a clustering method using the Gaussian mixture model to classify the never-smoker LUAD study cohort based on the expression of our novel cancer-immune gene panel. Although the study cohort was homogeneous in terms of tumor histology, smoking status, and gender, we identified three distinct subgroups that revealed differential expression of cancer-immune genes within TME, consisting of subgroup 1 (*n* = 37), subgroup 2 (*n* = 15), and subgroup 3 (*n* = 47) (Figures 1A and S4). This suggested immunological heterogeneity of our cohort despite similar clinical features. As heatmaps in the study cohort were obtained from the curatively resected tumors of patients with cancer who are treatment naive, we assumed that they represented the gene expression profile at the phase of immune escape in cancer immunoediting. Thus, subgroup-specifically altered gene expression could be regarded as the inherent cancer-immune status, equivalent to “cancer immune-set point” (Chen and Mellman, 2017) or “immune escape point.”

### Clinical and Pathological Features among Three Subgroups

To identify patient or tumor features that differentiated among subgroups, we compared clinical characteristics among the three subgroups. Compared with subgroup 1 and subgroup 3, patients in subgroup 2 tended to have relatively smaller tumors at the earlier stage of disease. However, there were no significant differences among the three subgroups in patient age, histologic subtype, tumor size, pathologic stage, tumor differentiation, or lymphovascular invasion (Figure 1B). Survival analyses also showed that there were no significant differences among the three subgroups in terms of overall and recurrence-free survival (Figure S2).

Next, the distribution of cellular components within TME were compared among subgroups, based on hematoxylin and eosin (H&E) histological examination. Stromal areas were predominantly occupied by stromal and other cells (macrophages with or without anthracotic pigments, neutrophils, and eosinophils) rather than lymphocytes, and a small number of lymphocytes were dispersed in stromal areas but not in tumor areas across the subgroups (Figure 1C). Accordingly, we did not observe any specific findings on H&E slides and T cell infiltration into tumor mass that could distinguish the three subgroups.

### Exome Analysis

**Figure 2:**
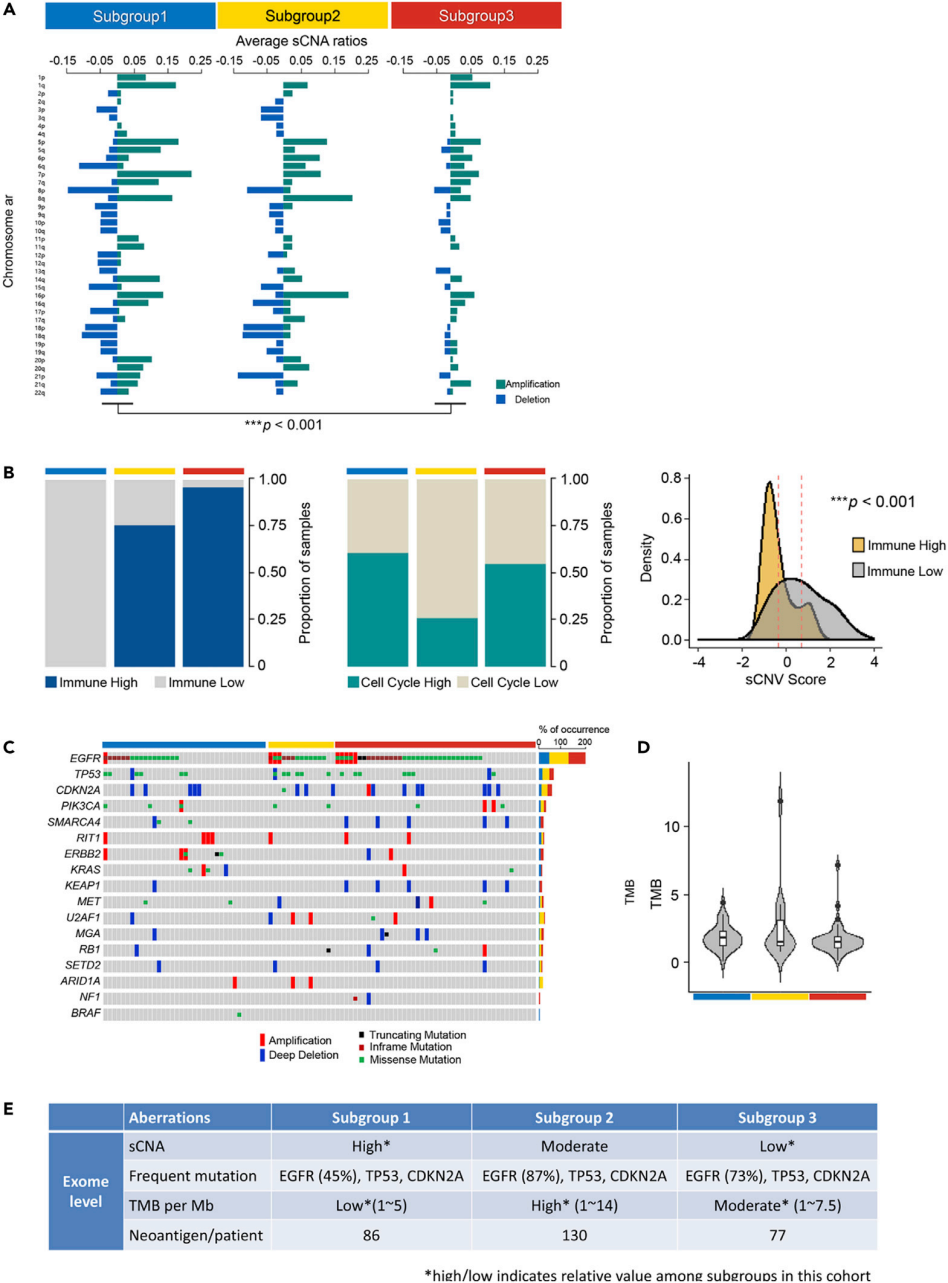
Exome-Level Subgroup Analysi (A) Somatic copy number variations. Somatic copy number variations (amplification or deletion) at the level of chromosome arms identified by GISTIC analysis were summarized for each subgroup. Each bar represents average of sCNA ratios across samples. ∗∗∗p<0.001, Student's t test. (B) Relationship of sCNV score with tumor proliferation and immune evasion. Bar charts show relative proportions between samples with high and low signature scores on two cancer hallmarks (immune and cell cycle signature). Higher- and lower-level samples were based on > 70^th^ and <30^th^ percentiles, respectively. Signature score was determined by the average gene expression level of each signature. Density plot showing distribution of sCNV scores, according to immune score. The x axis is normalized sCNV score across samples. (Immune High: −0.322(±0.127) and Immune Low: 0.704(±0.208)). ∗∗∗p<0.001, Student's t test. (C) Somatic mutations. From the whole-exome sequencing, 17 genes with most frequent somatic mutations were selected, identifying 5 different gene aberrations: amplification, deep deletion, truncating mutation, inframe mutation, and missense mutation. (D) Tumor mutation burden (TMB) shown for each subgroup. Every subgroup had TMB at the level of <5 per Mb (mode: ∼2 per Mb). Subgroups 1, 2, and 3 had the least, greatest (up to 14 per Mb), and intermediate (up to 7.5 per Mb) variations, respectively. Most patients, except the outliers with TMB exceeding 5 per Mb, were likely to have immune evasion due to the extremely low probability of immunogenic neoantigen generation. (Subgroup 1: 1.919(±0.146), Subgroup2: 2.644(±0.720), and Subgroup3: 1.700(±0.166).) (E) Major features at exome level are summarized. Average numbers of neoantigens in each subgroup were calculated as described in Methods (Table S1).

To determine whether any inter-subgroup differences at the genome level existed, whole-exome sequencing analysis was performed for the following three parameters: (1) somatic copy number alteration (sCNA); (2) somatic mutations, including missense, truncating, INDEL mutations; and (3) TMB. sCNA score was higher in subgroup 1 than in the other two subgroups (Figure 2A), and the higher sCNA score corresponded to a higher cell cycle signature score and lower immune signature score (p < 0.001 and p = 0.1, respectively; Mann-Whitney U test) (Figure 2B and Data S3), which is in good agreement with tumor survival via immune escape (Davoli et al., 2017).

Among the somatic mutations, *EGFR*, *TP53*, and *CDKN2A* had the most frequent mutations in descending order. *EGFR* mutation had the highest frequency across the cohort (45%–87%) and the most missense and inframe mutations, and amplifications, followed by *TP53* with missense mutations, and *CDKN2A* with deep deletions (Figure 2C); however, TMB level was very low (mode: ∼2 per Mb), with the highest variance in subgroup 1 (∼15 per Mb), followed by subgroup 3 (∼7.5 per Mb), and the lowest variance in subgroup 2 (Figure 2D). Thus, in this cohort, the high sCNA score and the low TMB level together with the strong molecular driver, *EGFR* mutation, appeared to contribute to the strong immune evasion (Figure 2E).

### Immune Cell Profile

**Figure 3:**
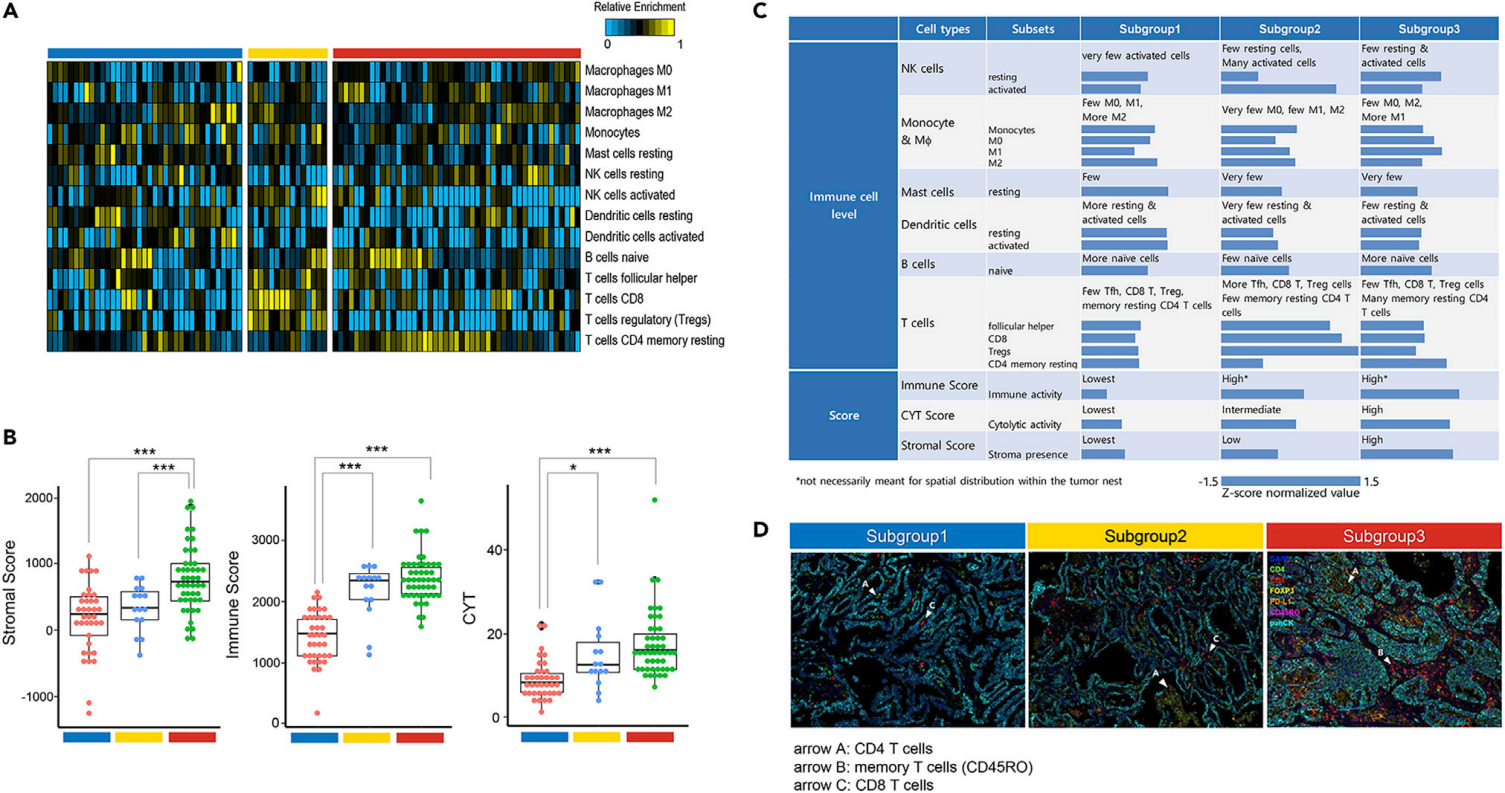
Subgroup Analysis of Immune Cell Profile by Whole-Transcriptome and Immunofluorescence Analyses (A) Subgroup-specific distribution of immune cell subsets shown after CIBERT calculation using RNA sequencing expression profiles, indicating heterogeneous frequencies for each subset. Prominence of CD8 T regulatory T cells in subgroup 2, and B cell naive and memory resting CD4 T cells in subgroup 3. Major features tabulated in Figure 3C. (B) Comparison of three subgroup-specific scores, namely, stromal, immune, and cytolytic activity scores (Data S3). Subgroups 1, 2, and 3 were the lowest, intermediate, and highest, respectively. Subgroup 2 had higher levels of CD8 T and regulatory T cells, yet had lower cytolytic activity scores than subgroup 3, consisting mostly of memory resting CD4 T cells. (∗p < 0.05; ∗∗p < 0.01; ∗∗∗p < 0.001, Student's t test.) (C) Most immune cells were not activated in subgroup 1. NK cell activation and high immune score were common features between subgroups 2 and 3. Subgroup 2 had relatively more CD8 T cells, Treg cells, and lower stromal score, whereas subgroup 3 had significantly more memory resting CD4 T cells and higher stromal score. A bar graph represents the quantification of the immune cell profile in each subgroup. Each proportion or score was normalized by *Z* score across samples, and then averages were taken for each subgroup. (D) Multiplexed immunofluorescence images showing distribution of T lymphocyte subsets, clearly distinguishable among subgroups. Very few CD8 T and CD4 T cells in subgroup 1. Subgroup 2 showing sparse CD8 T (arrow C) and CD4 T cells (arrow A) outside the tumor nests, and more CD4 T and memory T cells (CD45RO, arrow B) in subgroup 3 (cyan, pan-cytokeratin in tumor cells; green, CD4; red, CD8; yellow, FOXP3; orange, PD-L1; magenta, CD45RO; blue, DAPI).

To gain insight into the immune landscape and distinctive features in each subgroup, representative types of immune cells and their subsets were analyzed at the whole transcriptomic level (Figure 3A). Both immune score (the level of the infiltration of immune cells in tumor tissue) and stromal score (the level of stroma in tumor tissue) were the highest in subgroup 3 and the lowest in subgroup 1. Subgroup 2 was comparable to subgroup 3 in the immune score, whereas it was comparable to subgroup 1 in the stromal score (Figure 3B). In addition, subgroup 2 showed a relatively higher proportion of activated NK cells, CD8 T cells, and regulatory T cells (p = 7.7 × 10^−3^, p = 1.5 × 10^−5^, p = 3.1 × 10^−4^, respectively), which most probably resulted from aberrant editing of the peptide-MHC class I complex (Table 1). Furthermore, subgroup 3 showed a significantly higher proportion of memory resting CD4 cells (p = 3.6 × 10^−4^). However, the patterns of CYT, represented by GZMA and PRF1 expression of immune cells in subgroups, were found similar to the immune score pattern. This suggested that three patterns of immune transcriptome in never-smoker LUAD might be related to the proportions and types of constituent immune cells in the TME. Characteristics of the immune cell profile are summarized in Figure 3C, showing that immune score was more influenced by both TMB level and sCNA scale (Figure 2E).

**Table 1:** Major Features from the 8 Panels of Cancer-Immune Gene Expression

	Genes Associated	Subgroup 1	Subgroup 2	Subgroup 3
		UB+ IFN1p- AP-	UB- IFN1s- AP-	UB+ IFN1+ AP+
(De)Ubiquitination	Deubiquitination/E3 ubiquitin ligase	High	Very low^a^	High
Type I IFN	Production	Low	Very low	High^a^
	Signaling	High	Very low	High
Antigen-presenting machinery	Antigen processing	Low	Low	Low
	MHC class I	Low∗	High	Low
	MHC class II	Low	Low	High
	p-MHC assembly	High	ab. peptides^b^	High
	Antigen presentation	Very few p-MHC	MHC-I-bound ab. peptidome^b^	p-MHC-II
TGF-β	Signaling	High	Very low	High∗
NK cell activation	NKp30	Low	High	High
	NKp44	Low	Low	Low
	NKp46	Low	Low	High
	NKG2D	Low	Low	High
	NKG2A	Low	Low	High
	CD94	Low	Low	High
DC activation	Maturation	Low	High	High
	Migration to LN	Low	Low	High
TME	Myeloid cell recruitment	Low	High	High
	Angiogenesis	High	Low	Low
T cell activation (Treg included)				
neoAg-specific fraction	PD-1	Low	High	High
	CXCR3	Low	High	High
	IFNγ	Very low	Low	Low
Tumor-T cell interaction				
T cell responsiveness	IFNGR1	Low	Very low	High
	PD-L1	Low	Very low	High
	CXCL9/10/11	Very low	Low	High

Based on the major features, we temporarily renamed each subgroups as follows: subgroup 1 as subgroup with ubiquitination functioning, type I IFN production defective, antigen presentation defective (abbreviated as UB + IFN1p- AP-); subgroup 2 as subgroup with ubiquitination defective, type I IFN signaling defective, antigen presentation defective (UB- IFN1s- AP-); subgroup 3 as subgroup with ubiquitination functioning, type I IFN functioning, antigen presentation functioning (UB + IFN1+ AP+).

a High, low, or very low indicates relative value within this cohort.

b Aberrant peptides, aberrant peptidome.

### Cancer-Immune Gene Expression Analysis

To identify genes that play dominant roles in determining the immune transcriptome profile in each of three subgroups, gene expression levels were analyzed for each panel in the cancer-immunity cycle, and the possible cause-effect relationship was inferred between the expression of functionally associated genes in the pathway (Table 1 and Data S2).

#### Ubiquitination, Type I IFN Production/Signaling, and TGF-β Signaling

Post-translational modification processes ubiquitination and deubiquitination have been implicated in the regulation of innate and adaptive immune responses, which both play crucial roles during immune system development and stimulation of an immune response, ensuring proper functioning of immune cells (Hu and Sun, 2016). Therefore, any alteration in the ubiquitination system could affect signaling of cancer-immune genes, especially in the type I IFN pathway and TGF-β signaling panels of the cancer-immune cycle. Subgroup 2, unlike subgroups 1 and 3, expressed most ubiquitinating enzyme genes at an extremely low level, with concomitant low expression of genes involved in type I IFN production/signaling and TGF-β signaling pathway. Subgroup 1 showed downregulation of type I IFN production.

#### Antigen-Presenting Machinery

Subgroup 1 had significantly low expression of MHC classes I and II and overall antigen processing machinery, including B2M, indicating very poor presentation of tumor neoantigens, if any. Subgroup 2 showed unhindered expression of MHC class I, but low expression of peptide-trimming machinery (ERAPs and CANX) and MHC class II, which led to generation of the MHC class I-bound aberrant peptidome (Lopez de Castro, 2018). In contrast, most patients in subgroup 3 showed proper expression of MHC class II, but low expression of MHC class I.

#### NK Cell Activation

No activated NK cells were detected in subgroup 1. Subgroup 2 showed expression of an activated NK cell-activating receptor, NKp30. However, subgroup 3 showed activation of both types of receptors, i.e., activating receptors (NKp30, NK44, NK46, and NKG2D) and inhibitory receptors (NKG2A and CD94).

#### TME, DCs, and T Cell Activation

Based on examination of gene expression patterns between panels, the overall immune status of TME was influenced more by inflammation-related processes, including type I IFN signaling, antigen presentation, and NK cell activation, than by TGF-β signaling in this cohort (Figures 1A and S4). Subgroup 1 showed increased expression of pro-angiogenic factors (VEGFA and HIF1A), angiogenesis-related chemokines and their receptors, and the glutamate/glutamine transporter. Low expression of CCR7, CCL19, and CCL21 likely led to extremely low levels of DC maturation and inefficient trafficking of lymphocytes to lymph nodes. Subgroup 2, in contrast, showed increased expression of transcription factor BATF3 of DCs and chemotactic factors (CCL19, CCL21, CXCL13) of naive T cells, central memory T cells, and B cells in lymph nodes. Expression levels of molecules for T cell maturation and activation (CD2, CD27, PDCD, ZAP70, LAT, LCK), endosome recycling (RAB4B, RAB11B), and costimulation of T cells, including follicular helper T cells and regulatory T cells (TNFRSF4 [OX40], TNFRSF14 [LIGHT ligand receptor], TNFRSF18 [GITR]), were upregulated. Subgroup 3 had less immune suppressive TME compared with the other subgroups with functional DC maturation and migration, and most genes related to T cell maturation and activation were upregulated.

Subgroups 2 and 3 showed similar activation patterns of T cells (i.e., low IFNγ, high PD-1, and CXCR3), whereas significant differences existed in the subsets of activated T cells (Figure 3C). Furthermore, significant differences occurred between subgroups 2 and 3 at the level of IFNγ receptor expression on tumors, which rendered tumor cells sensitive to IFNγ.

### Coherence of Gene Expression, Immune Cell Profile, and Multiplexed Immunofluorescence Findings

Internal consistencies were examined between cancer-immune gene expression at the panel level and scores from the immune cell profile such as stromal score, immune score, and CYT from the whole transcriptome analysis (Figure 3C). A high stromal score indicated the presence of inflammation and angiogenesis in the system, which is most influenced by type I IFN production/signaling (Liu et al., 2018). Thus, subgroup 1 had the lowest stromal score, whereas subgroup 3 had the highest, which agrees with the immune cell profile.

Immune score usually refers to the immune cell density in the tumor mass (Galon et al., 2012). Therefore, with the immune score only, it is difficult to predict spatial distribution of immune cells with respect to the tumor and stromal cells (Kather et al., 2018). This was confirmed by our observation that subgroups 2 and 3 had relatively high and comparable immune scores, but the expression patterns of genes along the pathway leading to T cell activation and the distribution of T cells and their subsets were quite different (Figures 1, 3A, and 3C).

CYT usually refers to cytotoxic immune cells that contain perforin and granzyme B. Subgroup 2 had an intermediate CYT score with activated NK cells, CD8 T cells, and regulatory T cells, whereas subgroup 3 had the highest CYT score with mostly memory resting CD4 T cells as cytotoxic immune cells. As a result, subgroup 2 seemed to have MHC class I-bound aberrant peptidome due to aberrant editing of antigenic peptides, activating regulatory T cells with T cell receptor (TCR) to suppress the aberrant neoantigen repertoire (Akkaya et al., 2019; Weissler and Caton, 2014). Conversely, subgroup 3 had normal expression of MHC class II, but low level of TMB and low expression of MHC class I molecules, which would result in rapid clearance of the clonal fraction of neoantigen-containing tumor cells by the resultant neoantigen-specific cytotoxic CD4 T cells, even in small number (Takeuchi and Saito, 2017), and subsequent conversion to memory resting cells with perforins and granzyme B still retained (Lin et al., 2014).

This immune cell profile agreed with the multiplexed immunofluorescence images regarding spatial distribution of lymphocyte subsets in or around the tumor mass, such that very few lymphocytes were detected in subgroup 1, even in the stromal area. Subgroup 2 expressed slightly more T cells, and subgroup 3 had a relative abundance of memory CD4 T cells, mostly positioned in the stromal area with a low level of tumor infiltration (Figure 3D). As the memory resting CD4 T cells were likely to have been induced by the MHC-II-carrying tumor cells in subgroup 3 (Table 1), and could be reactivated to cytotoxic CD4 T cells through contact with tumor cells possessing the corresponding epitopes (MacLeod et al., 2010), potential ICB responders could be found in this subgroup of never-smoker patients with LUAD.

### Subgroup Renaming

Major features from the eight panels of analysis of cancer-immune gene expression are summarized in Table 1. Considering the function or defectiveness in ubiquitination, type I IFN production/signal, and antigen-presenting machinery, subgroups were renamed accordingly. For example, subgroup 1 was renamed as subgroup (UB+ IFN1p- AP-), subgroup 2 as subgroup (UB- IFN1s- AP-), and subgroup 3 as subgroup (UB+ IFN1+ AP+).

### T Cell Infiltration and Tumor Killing

It remains unclear how discrepancies in upstream processes of the cancer-immunity cycle (as seen in the new subgroup names) would determine the modes of T cell activities and behaviors such as tumor infiltration, tumor recognition, and tumor killing (Trujillo et al., 2018). Therefore, the activation status of T cells and tumor cells were compared, focusing on seven factors, i.e., NK cell-activating receptors from NK cells, PD-1, CXCR3, and IFNγ from T cells, and IFNγ receptor, PD-L1, and CXCL9 from the tumor, of which the last six factors are directly involved in the activation and trafficking of T cells and their interaction with tumor cells (Table 1). Overall, it was not possible to see a visible difference in IFNγ expression; however, significant differences in expression levels of the other five factors among subgroups were found. Furthermore, the pattern of PD-1 and CXCR3 expression level as markers of T cell activation status seemed similar to that of DC maturation status and NK cell-activating receptor (NKp30), which implies that activated NK cells can be an indicator of DC maturation and T cell activation.

From these results, high expression levels of NK cell-activating receptors, activated T cell markers (PD-1, CXCR3, and especially IFNγ), and tumor's T cell recruitment-associated markers (IFNγ receptor, PD-L1, and CXCL9) were necessary for successful T cell infiltration and tumor killing (Higgs et al., 2018). This study confirmed that IFNγ receptor was expressed at a high level in subgroup (UB+ IFN1+ AP+), with proper functioning of type I IFN signaling and NK cell activation.

### Subgroup Enriched with ICB Responders

**Figure 4:**
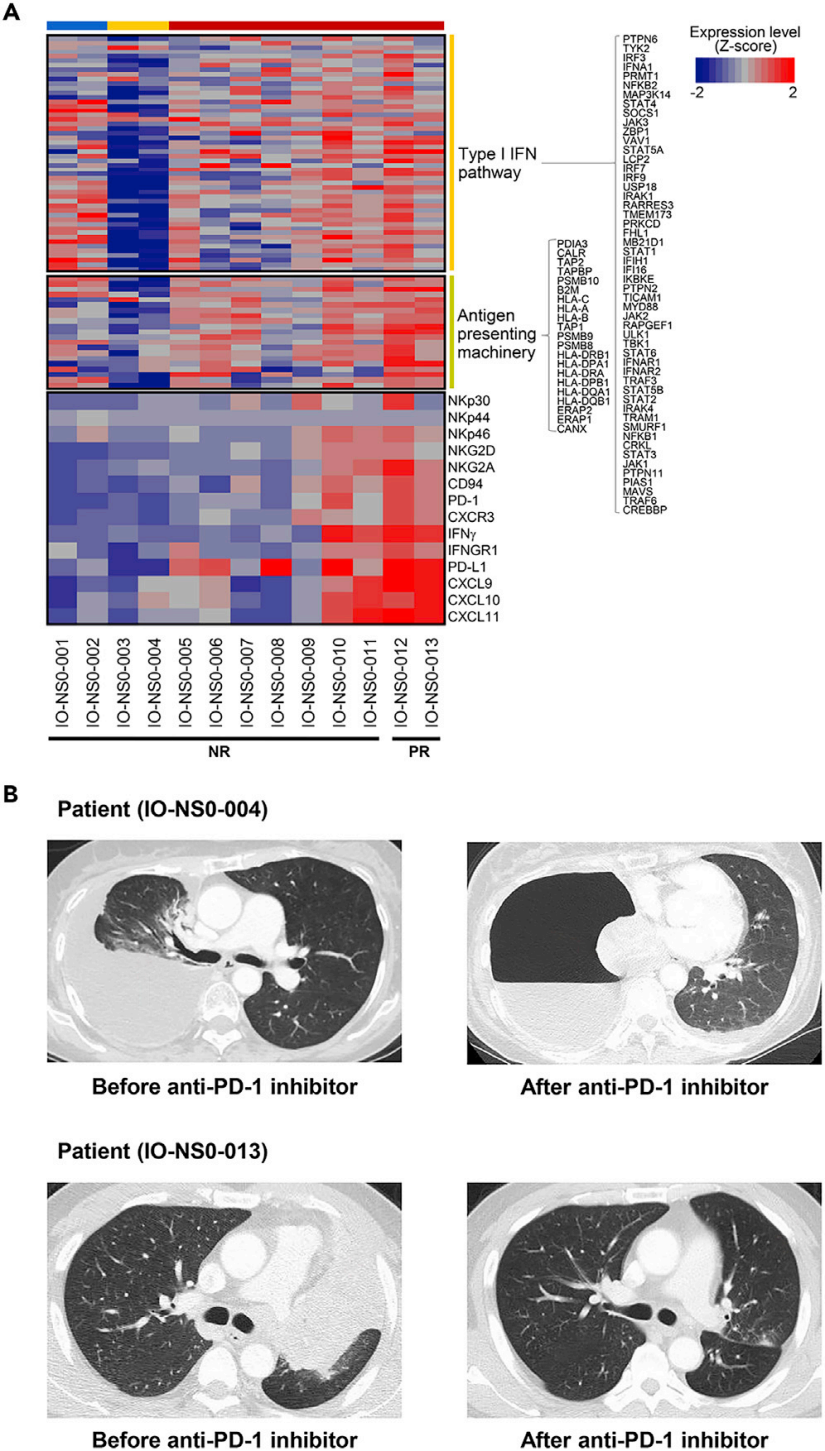
Expression of Selected Cancer-Immune Genes Determining Tumor-T Cell Interaction in Independent Cohort (A) Expression of genes determining tumor-T cell interaction. Biopsied samples from independent cohort of naive 13 never-smoker patients with LUAD were analyzed for cancer-immune gene expression, and their transcriptomic patterns of genes related to type I IFN production/signaling, antigen-presenting machinery, NK cell activation, T cell activation, and tumor readiness for T cell recruitment were compared with ICB response. Patients with partial response (IO-NS0-012 and -013) were found only in subgroup (UB+ IFN1+ AP+), which fits well with the finding from the study cohort. From the four cases (IO-NS0-010, IO-NS0-011, IO-NS0-012, IO-NS0-013), the concerted expression of six genes such as PD-1, CXCR3, IFNγ, IFNGR1, PD-L1, and CXCL9 was necessary for the successful response to ICB. NR: non-response; PR: partial response. (B) Computed tomographic (CT) imaging before and after ICB administration for the two representative cases. In the upper row, a 57-year-old never-smoker female (IO-NS0-004; subgroup [UB- IFN1s- AP-]) with stage IV *EGFR*-mutant LUAD (19 deletion) metastasizing to pleura, brain, bone, and liver received two doses of nivolumab; CT image showed progressive disease with her partially atelectatic right lung totally collapsed due to tumor invading the right main bronchus. In the lower row, a 59-year-old never-smoker male (IO-NS0-013; subgroup [UB+ IFN1+ AP+]) with stage IV LUAD with *ALK* translocation received two doses of pembrolizumab and then showed a partial response with a complete resolution of total atelectasis of the left upper lobe.

Based on the analysis, subgroup (UB+ IFN1+ AP+) likely represents potential ICB responders. To confirm, 13 independent never-smoker patients with LUAD who had ICB treatments were studied. In brief, whole-exome/transcriptome data were obtained from their snap-frozen tumor tissues, which were stratified into three subgroups using Gaussian mixture model clustering. The patterns in cancer-immune gene expression in three subgroups including a subgroup with ubiquitination defective in this independent cohort were found to be very similar to those in the study cohort. Subsequently, we compared the clinical efficacy determined by the immune response evaluation criteria in solid tumors (irRECIST) with subgroup-specific patterns of cancer-immune gene expression (Figures 4A and 4B) (Hodi et al., 2018). In particular, we focused on seven major factors (as mentioned in the previous section) of T cell infiltration and tumor killing (Figure 4A). Two of nine patients (22%) in subgroup (UB+ IFN1+ AP+) had an initial reduction in tumor size by 30% or more (partial response). This strongly supported that potential ICB responders very likely belonged to this particular subgroup in never-smoker LUAD. As such, Gaussian mixture model clustering based on the cancer-immune gene panel provided information about the subgroup enriched for the active cancer-immunity cycle without any prior knowledge of the immune escape points.

From those two partial response cases in this subgroup, we observed that tumor-specific T cells effectively infiltrated and removed tumor cells upon ICB treatment when patients with activated NK cells (i.e., high level of NKp46) had both high expression of IFNγ from activated T cells (i.e., high level of PD-1 and CXCR3) and PD-L1 and CXCL9 from tumors (high level of IFNγ receptor). Taking further into account the individual heterogeneity present in the subgroup and the larger-scale study, it would be possible to develop more precise patient selection signatures for ICB treatment.

## Discussion

From our approach using the cancer-immune gene expression panels, we could classify the cohort of never-smoker patients with LUAD with clinicopathological homogeneity and very low level of TMB into three subgroups. Furthermore, we found that in 15% of the cohort, as a distinct subgroup (UB− IFN1s− AP−), there was significant downregulation of the ubiquitination system, demonstrating a significant impact on type I IFN and TGF-β signaling.

**Figure 5:**
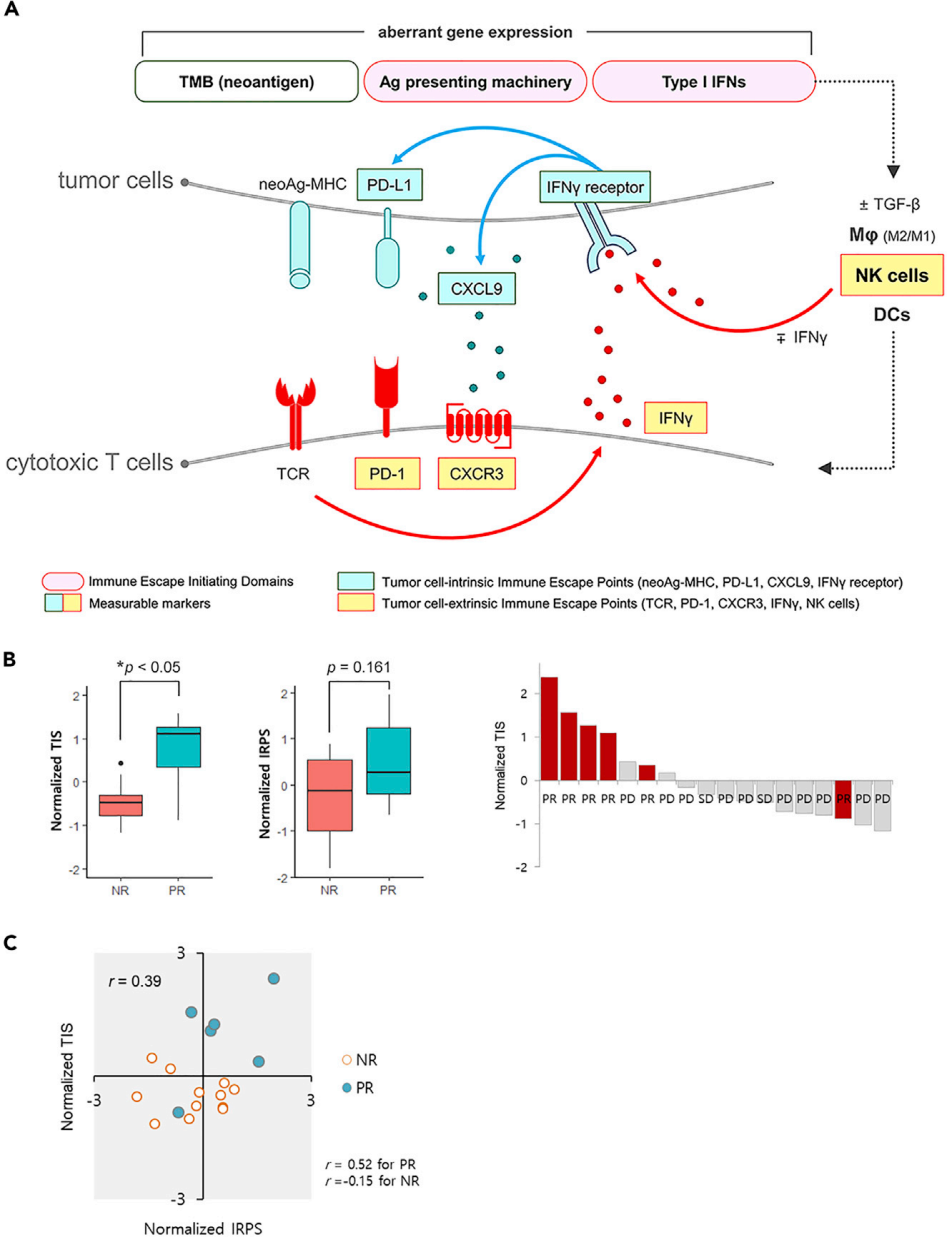
Schematic Model for Major Immune Escape-Initiating Domains and Immune Escape Points in Tumor-T Cell Interaction (A) Major immune escape points depicted in schematic model, highlighting tumor-T cell interaction at last stage of cancer-immunity cycle. Briefly, tumor cells express neoantigen as neoantigenic peptide-MHC and IFNγ receptor via antigen-presenting machinery (APM) and type I IFN (IFN1) pathway, respectively. Further, TGF-β levels in the tumor microenvironment determine macrophage polarization. Concurrently, activated macrophages (M1 type) in the proximity of tumor cells activate IFNγ-secreting NK cells, leading to IFNγ receptor activation on tumor cells. In response, tumor cells stimulate PD-L1 expression and CXCL9 chemokine secretion. The activated NK cells enrich mature DCs, which migrate to lymph nodes, activating and proliferating T cells with surface PD-1, CXCR3, and neoantigen-specific T cell receptors. The activated T cells migrate through the vascular system, extravasate, and infiltrate into the tumor microenvironment for tumor-T cell interaction. When neoantigenic peptide-MHC-TCR binding occurs, T cells secrete IFNγ and initiate tumor killing. However, when macrophages remain inactivate (M2 type), and NK cells are not activated, tumor-T cell interaction does not occur. Thus, appropriate immune responses (such as T cell-mediated tumor killing) require at least nine activated molecules in the cancer-immunity cycle, which only occurs with properly functioning APM and IFN1 pathways in neoantigen-containing tumor cells. Functional alterations in the APM and/or IFN1 pathway (i.e., immune escape initiating domains) result in immune evasion. Therefore, these nine molecules are regarded as major immune escape points (7 points in the boxes are measurable). (B) To obtain the evidence for an important role of immune escape initiating domains in determining the immune response, Gene Expression Omnibus database GSE93157 was used as an independent cohort. Normalized TICs or IRPs for patients within the cohort were plotted against ICB responses. ∗p<0.05, Student's t test. (C) For another line of evidence, the normalized TICs were plotted against the normalized IRPs. NR: no response; PR: partial response; PD: progressive disease.

We also noted that one structural domain, TMB (neoantigens), and two functional domains, type I IFN production/signaling and antigen presentation machinery, were important determining factors for the eventual immune response in the tumor-T cell interactions. In this study, we named the functional domains as the immune escape-initiating domains because significant alterations in genes or gene expression in these domains at the early stage of the cancer-immunity cycle resulted in significant impacts on tumor-T cell interactions (i.e., immune escape points) due to unsuccessful binding between neoantigenic peptide-MHC and TCR, including immune evasion, which enabled uncontrolled proliferation of tumor cells. This inference was supported by two observations in the ICB-treated independent cohort, i.e., presence of ICB responders in subgroup (UB+ IFN1+ AP+), even if they had low TMB levels and the expression patterns of genes involved in tumor-T cell interactions (Figure 4A). Based on these observations of ours and other groups, a schematic illustration of this model is shown in Figure 5A, with an emphasis on the immune escape-initiating domains and their eventual effect on the immune escape points in tumor-T cell interactions.

In addition to TMB (neoantigens), the nine immune escape points derived from the two immune escape-initiating domains were found to be critically important for the anti-cancer immune outcome. First, the type I IFN production/signaling pathway stimulates recruitment and activation of innate immune cells, including macrophages, NK cells (Muller et al., 2017), and DCs, aiding IFNγ secretion by NK cells (Reefman et al., 2010). Tumor cells express PD-L1 and secrete CXCL9 upon IFNγ receptor binding to IFNγ (Ayers et al., 2017). Second, antigen-presenting machinery together with the size and expression level of the antigen repertoire are determining factors for the antigenic peptide-MHC complex in tumor cells, which, with the help of activated NK cells and matured DC, lead to the generation of neoantigen-specific activated T cells expressing PD-1, CXCR3, and IFNγ. The activated T cells infiltrate tumors through chemokine CXCL9-mediated migration to kill the tumor cells.

From this model, we can deduce that the level of PD-L1 expression is modulated in the following context: (1) any alteration in the pathway leading to expression of PD-L1 or its post-translational modification (Hsu et al., 2018), (2) any alteration in the IFNγ signaling pathway including expression of IFNγ receptor or its post-translational modification (Gao et al., 2016; Garcia-Diaz et al., 2017), (3) IFNγ-stimulated chemokine CXCL9 secretion from tumor cells through M1 macrophage-activated NK cells in the inflammatory TME (Bellora et al., 2010), and (4) clonal fraction of tumor cells with neoantigen-MHC and cytotoxic T cells with neoantigen-specific TCR. This could be the underlying reason why PD-L1 cannot be the absolute predictive marker for ICB treatment. Furthermore, low CXCL9 levels due to M2-polarized macrophages could not induce T cell infiltration, resulting in the immune-excluded phenotype (Peranzoni et al., 2018); thus, manipulation of TGF-β levels and/or its signaling in the TME for macrophage polarization could potentially increase the efficacy of ICB treatment (Nunez et al., 2018). All these phenomena suggest that the type I IFN pathway and antigen presentation are crucial for appropriate tumor-T cell interactions, which require both innate and adaptive immune processes.

To validate the working hypothesis that genes from the type I IFN pathway and antigen-presenting machinery are crucial for tumor-T cell interaction and determining ICB responses, we need an independent cohort of patients with LUAD who never smoke with the ICB response and gene expression data. However, such data are currently not available. Instead, as an alternative, we used the Gene Expression Omnibus database GSE93157, which contains information of 18 patients with melanoma who never smoke with their ICB responses and gene expression data from the PanCancer 730-Immune Panel (Prat et al., 2017).

For the representative values of the expression levels of genes in the type I IFN pathway and antigen-presenting machinery, we selected three genes (*IFI16, MYD88,* and *JAK2*) from the type I IFN pathway and four genes (*TAP1, TAP2, PSMB9,* and *HLA-DQA1*) from the antigen-presenting machinery and termed this group as a tumor-intrinsic classifier (TIC). Similarly, for the representative values of the expression levels of genes involved in tumor-T cell interaction, we selected six genes (*IFNGR1, PD-L1, CXCL9, IFNγ, PD-1,* and *CXCR3*) and termed this group as an immune response predictor (IRP). The expression levels of these 13 selected genes were found to be strongly correlated with the ICB response in patients with LUAD who never smoke (Figure 4A). Next, we calculated the geometric means of the expression levels of the TIC and IRP, independently, for each patient, and then normalized the values within the cohort.

We observed a distinctive contribution of the normalized TIC to ICB response, compared with that of the normalized IRP alone, whereby higher normalized TIC had a more distinctive ICB response (Figure 5B, Data S4). Furthermore, we found that with a high Pearson's correlation coefficient, *r* = 0.52, four of six ICB responders were clustered in the first quadrant of the normalized TIC and IRP planes (Figure 5C, Data S5). This supports our working hypothesis that the type I IFN pathway and antigen-presenting machinery (which are major factors of the immune escape-initiating domain in patients with LUAD who never smoke) are dominant contributors to tumor-T cell interaction and ICB response, downstream of the cancer-immunity cycle, through causal relation in never-smoker patients with melanoma as well. We could not observe this relationship in other patients with cancer who smoke (Data S6).

In summary, we performed in-depth analyses of early-stage never-smoker female patients with LUAD to understand the immune context within TME. Using our novel panels of cancer-immune genes reflecting the immune landscape based on the cancer-immunity cycle, we identified three distinct subgroups in immune escape pathways in terms of the immune escape-initiating domains and proposed the immune escape points involved in tumor-T cell interactions in this clinically homogeneous cohort. Our approach could play a critical role in understanding the immune evasion mechanism at the individual level, developing patient selection signatures, and finding new targets or treatment strategies.

### Limitations of the Study

Single-cell RNA sequencing, spatial transcriptomic analysis, and comprehensive selection of cancer-immune genes would be needed for more detailed understanding of biological and geographical interaction between tumor cells and innate and adaptive immune cells at the level of gene expression. Information on the ICB response of patients with LUAD who do not smoke on a large scale should be accumulated to provide further substantial evidence of our tentative conclusion that early-stage cancer-immune gene expression determines the cancer immunity. It remains to be addressed whether distinct immune escape patterns observed in the study cohort would be specific for the female patients with LUAD who do not smoke, or could also be observed in male patients with LUAD or other patients with cancer who do not smoke.

### Resource Availability

#### Lead Contact

Further information and requests for resources should be directed to and will be fulfilled by the Lead Contact, Jhingook Kim (jhingookkim@gmail.com).

#### Materials Availability

This study did not generate new unique reagents.

#### Data and Code Availability

The data presented in this article have been deposited in NCBI's Gene Expression Omnibus and are accessible through GEO Series accession number GSE110907.

## Methods

All methods can be found in the accompanying Transparent Methods supplemental file.

## Supplemental Information

Document S1. Transparent Methods, Figures S1–S4, and Table S1

Data S1. Compilation of Panel Gene Sets and Their References, Related to Figure 1

Data S2. Characteristic Expression of Genes in Each Subgroup of Never-Smoker Female Patients with LUAD, Related to Table 1

Data S3. Scores Associated with Cancer Immunity and Immune Checkpoint Expression in Early-Stage Never-Smoker Female Patients with Lung Adenocarcinoma, Related to Figure 2

Data S4. Relationship between Normalized TIC and Immune Response in Patients with Melanoma Who Do Not Smoke, Related to Figure 5B

Data S5. Relationship between Normalized TIC and Normalized IRP in Patients with Melanoma Who Do Not Smoke, Related to Figure 5C

Data S6. Relationship between Normalized TIC and Normalized IRP in Various Patients with Cancer Who Smoke, Related to Figure 5C
