# Supplementary material for: Earlier-Phased Cancer Immunity Cycle Strongly Influences Cancer Immunity in Operable Never-Smoker Lung Adenocarcinoma
Source: iScience. 2020 Jul 18;23(8):101386. doi: 10.1016/j.isci.2020.101386 (PMC7426575; doi:10.1016/j.isci.2020.101386)
Supplement: Document S1. Transparent Methods, Figures S1–S4, and Table S1 [file mmc1.pdf]

iScience, Volume *23*

Supplemental Information

Earlier-Phased Cancer Immunity Cycle Strongly Influences Cancer Immunity in Operable Never-Smoker Lung Adenocarcinoma

Hong Kwan Kim, Je-Gun Joung, Yoon-La Choi, Se-Hoon Lee, Byung Jo Park, Yong Soo Choi, Daeun Ryu, Jae-Yong Nam, Mi-Sook Lee, Woong-Yang Park, Soohyun Hwang, Hongui Cha, Hong Sook Kim, Sanghyuk Lee, Yeonjoo Jung, Jong Eun Lee, Junsang Doh, Soonmyung Paik, Jung Hee Kang, Jinseon Lee, and Jhingook Kim

Supplemental Information

Earlier-phased cancer immunity cycle strongly influences cancer immunity in operable never-smoker lung adenocarcinoma

Kim et al.

TRANSPARENT METHODS

Patients

Eligible patients had stage I, II, or IIIA NSCLC deemed surgically resectable before enrollment. This cohort consisted of never-smoker female patients who underwent curative-intent surgery for LUAD (Figure 1C). Most patients had stage I (*n* = 62) or II (*n* = 16) disease. Additionally, 21 patients with stage IIIA were included in the cohort. and 5 of which (23.8%) received neoadjuvant concurrent chemoradiotherapy. All patients, except one, achieved complete tumor removal during surgery. Patient samples were obtained from LUAD patients who had undergone curative surgery in the Samsung Medical Center. All samples and clinical information from medical records were collected with patient written informed consent. This study was approved by the Institutional Review Boards of Samsung Medical Center (IRB no. 2010-08-063-006) in accordance with the Declaration of Helsinki.

Exome- and transcriptome-seq sample preparation and sequencing

RNA purity was determined by assaying 1 L of the total RNA extract on a NanoDrop ND-1000 spectrophotometer (ThermoFisher, Waltham, MA, USA). Total RNA integrity was assessed using a Bioanalyzer 2100 with an RNA Integrity Number value greater than 8 (Agilent, Santa Clara, CA, USA). Subsequently, mRNA sequencing libraries were prepared according to the manufacturer's instructions using the Illumina Truseq RNA Prep kit v2. The quality of the amplified libraries was verified using an Agilent Bioanalyzer 2100. Sequencing of pooled libraries was performed on the HiSeq 2000 sequencing system with paired-end reads of 100 bp length (Illumina, San Diego, CA, USA). Deep sequencing data were deposited in the Gene Expression Omnibus (GSE110907) database. Whole exome sequencing of tumors and matched normal blood samples was performed as described in our previous study (Kim et al., 2013).

Exome sequence data analysis

Sequencing reads were aligned to the University of California Santa Cruz hg19 reference genome (downloaded from http://genome.ucsc.edu) using the Burrows-Wheeler Alignment tool (BWA) v. 0.6.2 with default settings (http://bio-bwa.sourceforge.net/). PCR duplicate reads were marked using Picard-tools-1.8 (http://picard.sourceforge.net/), and data clean-up steps were performed using GATK-2.2.9 (https://software.broadinstitute.org/gatk/). Somatic point mutations were identified with the MuTect tool (https://github.com/broadinstitute/mutect) in paired samples and were annotated by ANNOVAR (http://annovar.openbioinformatics.org/).

RNA-sequence data analysis

Reads from the FASTQ files were mapped to the hg19 human reference genome, using STAR version 2.5.0a in 2-pass mode (https://github.com/alexdobin/STAR), and gene quantification was performed using RSEM (RNA-Seq by Expectation Maximization) (https://deweylab.github.io/RSEM/). Expressed genes were defined as a transcript per million (TPM) value of more than 10 across all samples to reduce the false positive rate. Stromal and immune scores based on the transcriptome were calculated using ESTIMATE. Fractions of immune-associated cell types were calculated by CIBERSORT (https://cibersort.stanford.edu/) using RNA-seq expression profiles. The immune CYT score was measured by taking the geometric mean of *GZMA* and *PRF1* expression values in TPM (Rooney et al., 2015).

Calculation of SCNA

Copy number variations were detected using EXCAVATOR software (https://omictools.com/excavator-tool). Significantly recurrent somatic copy number alterations were identified by GISTIC analysis (Mermel et al., 2011) with previously applied parameter values: a noise threshold of 0.3, a broad length cutoff of 0.5 chromosome arms, a confidence level of 95%, and a copy-ratio cap of 1.5 (Zack et al., 2013). Arm and focal SCNA levels of each patient were calculated by summing the copy number alterations (Davoli et al., 2017). Arm-level SCNAs and formal-level SCNVs were determined by length > 98% and length < 98% of each chromosome, respectively.

TMB and prediction of candidate neoantigens

TMB was measured by the number of somatic single nucleotide variants and indel

mutations per megabase in the coding region (Chalmers et al., 2017). Somatic single nucleotide variants included nonsynonymous and synonymous mutations. Non-coding alterations were not counted.

Neoantigens were predicted using MuPeXI v.1.1.3 (https://github.com/ambj/MuPeXI/). The three types of human leukocyte alleles (HLA-A, -B, and -C) were identified from the RNA-seq data of each patient using seq2HLA (https://bitbucket.org/sebastian_boegel/seq2hla). Somatic mutations, gene expression counts, HLA types for each patient, and peptide lengths (8-11 mer) were provided as input for MuPeXI. Peptides with a half maximal inhibitory concentration (IC50) value ≤ 500 nM were considered to have a high binding affinity for the MHC. The top 2% ranked neoantigens were finally selected.

Identification of subgroups based on Gaussian mixture models

Patient subgroups were determined using the Gaussian mixture model. Gene expression profiles of cancer immune pathways were clustered by mclust (https://cran.r-project.org/package=mclust), model-based clustering based on finite Gaussian mixture models. The initial partitions were performed by hierarchical model-based agglomerative hierarchical clustering. Following, the models were estimated by the expectation-maximization (EM) algorithm. The optimal model selection was based on Bayesian information criterion (BIC). Genes belonging to each functional category were ordered by hierarchical clustering.

Histologic examination and quantitative image analysis

Hematoxylin and eosin (H&E) staining was performed on all paraffin blocks with tissue samples, and an H&E slide from each case was selected and scanned using the Vectra Polaris quantitative slide scanner (PerkinElmer). Phenochart (PerkinElmer, Ver. 1.0.9) was used to view whole slide images and extract at least 10 regions of interest (ROIs) per sample. The size of each ROI was 682 x 510 µm. Images were analyzed using inForm software (PerkinElmer, Ver. 2.4). For tissue segmentation, the software was trained to automatically segment each image into tumor, stroma, and non-tissue regions. Cell segmentation to identify individual cells was conducted based on nuclear segmentation using hematoxylin counterstain as a parameter of nuclear component. Based on morphology, the following phenotypes were assigned to segmented cells for training: tumor, lymphocytes, stromal cells, and others (e.g. macrophages, neutrophils, and eosinophils). Misclassified cells were corrected in consecutive rounds of training, until at least 30 cells in each phenotype were selected for training. Cell segmentation data were then exported from inForm software as .txt files and processed using TIBCO Spotfire (PerkinElmer). Only cells with phenotype confidence level, which is the probability of the winning phenotype reported by inForm, of > 80% were included in the analysis.

Multiplexed immunofluorescence

Multiplexed immunofluorescence (IF) staining was performed using the Opal 7 Solid Tumor Immunology Kit (PerkinElmer), according to the manufacturer's protocol. In brief, formalin-fixed paraffin-embedded tissue slides were deparaffinized with xylene and rehydrated. Antigen retrieval was performed using AR9 or AR6 buffer and microwave treatment. Following incubation with the first antibody CD45RO (clone UCHL1), the secondary antibody

was incubated using Opal Polymer HRP. Opal 690 dye was used for visualization of CD45RO, followed by microwave treatment to remove primary and secondary antibodies. The process was repeated in the following order of antibodies/fluorescent dye: FOXP3 (clone 206D)/Opal 650, CD8 (clone 4B11)/Opal 620, PD-L1 (clone E1L3N)/Opal 570, CD4 (clone EP204)/Opal 540, pan-cytokeratin (clone AE1/AE3)/Opal 520. DAPI was applied to visualize nuclei, and multiplexed slides were imaged using the PerkinElmer Vectra Polaris quantitative slide scanner. The scanned images were analyzed using Inform software (PerkinElmer, Ver. 2.4.1).

Quantification and statistical analysis

Statistical analyses were performed using R v.3.1.2 software (https://www.r-project.org/). *P* values were derived from two-tailed tests, and those less than 0.05 were considered statistically significant.

REFERENCES

Chalmers, Z.R., Connelly, C.F., Fabrizio, D., Gay, L., Ali, S.M., Ennis, R., Schrock, A., Campbell, B., Shlien, A., Chmielecki, J., et al. (2017). Analysis of 100,000 human cancer genomes reveals the landscape of tumor mutational burden. Genome Med. 9, 34.

Kim, S.C., Jung, Y., Park, J., Cho, S., Seo, C., Kim, J., Kim, P., Park, J., Seo, J., Kim, J., et al. (2013). A high-dimensional, deep-sequencing study of lung adenocarcinoma in female never-smokers. PLoS One 8, e55596.

Mermel, C.H., Schumacher, S.E., Hill, B., Meyerson, M.L., Beroukhim, R., and Getz, G. (2011). GISTIC2.0 facilitates sensitive and confident localization of the targets of focal somatic copy-number alteration in human cancers. Genome Biol. 12, R41.

Rooney, M.S., Shukla, S.A., Wu, C.J., Getz, G., and Hacohen, N. (2015). Molecular and genetic properties of tumors associated with local immune cytolytic activity. Cell 160, 48-61.

Zack, T.I., Schumacher, S.E., Carter, S.L., Cherniack, A.D., Saksena, G., Tabak, B., Lawrence, M.S., Zhsng, C.Z., Wala, J., Mermel, C.H., et al. (2013). Pan-cancer patterns of somatic copy number alteration. Nat. Genet. 45, 1134-1140

Figure S1. Unsupervised clustering of never smoker lung adenocarcinoma by T-cell signature according to Gajewski's method, Related to Figure 1

A. Consensus clustering of 3,095 genes correlated with T-cell signature (CD8A, CCL2, CCL3, CCL4, CXCL9, CXCL10, ICOS, GZMK, HLA-DMA, HLA-DMB, HLADOA, and HLA-DOB).

B. Principal component analysis (PCA) of three distinct subtypes.

C. Expression patterns of 12 T-cell signature genes.

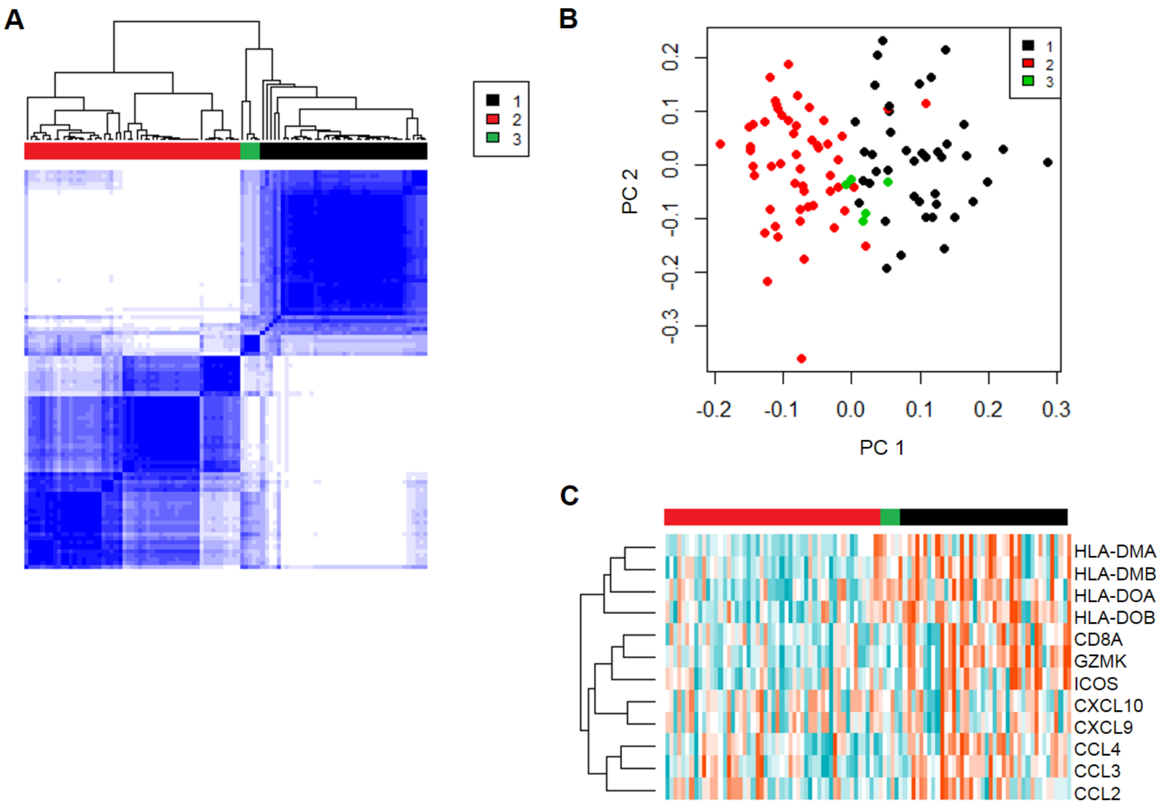

Reference:

Sweis RF, Spranger S, Bao R, Paner GP, Stadler WM, Steinberg G, Gajewski TF. Molecular Drivers of the Non-T-cell-Inflamed Tumor Microenvironment in Urothelial Bladder Cancer. Cancer Immunol Res. 2016 Jul;4(7):563-8.

Figure S2. Comparison of overall and recurrence-free survival between subgroups, Related to Figure 1

Overall Survival

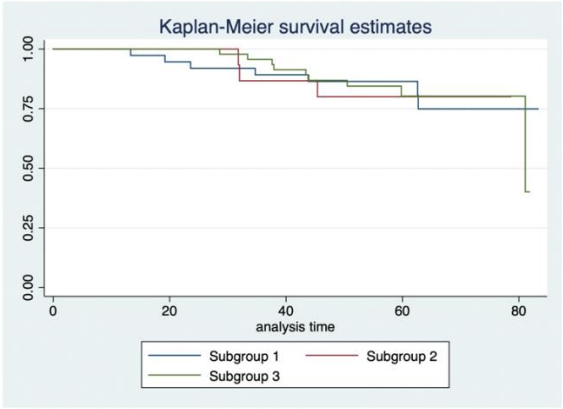

p = 0.944

Recurrence-free Survival

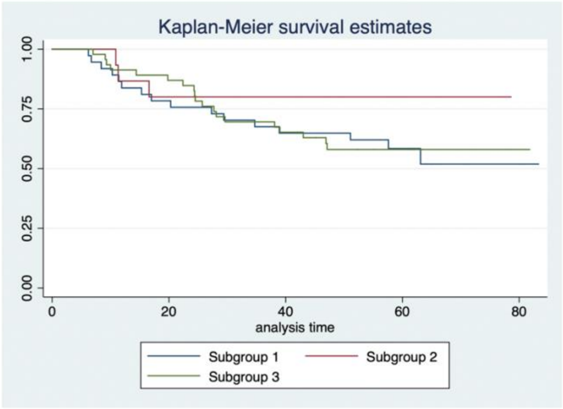

p = 0.374

Figure S3. Gene panels in this study superimposed on the Chen & Mellman's cancer-immunity cycle, Related to Figure 1

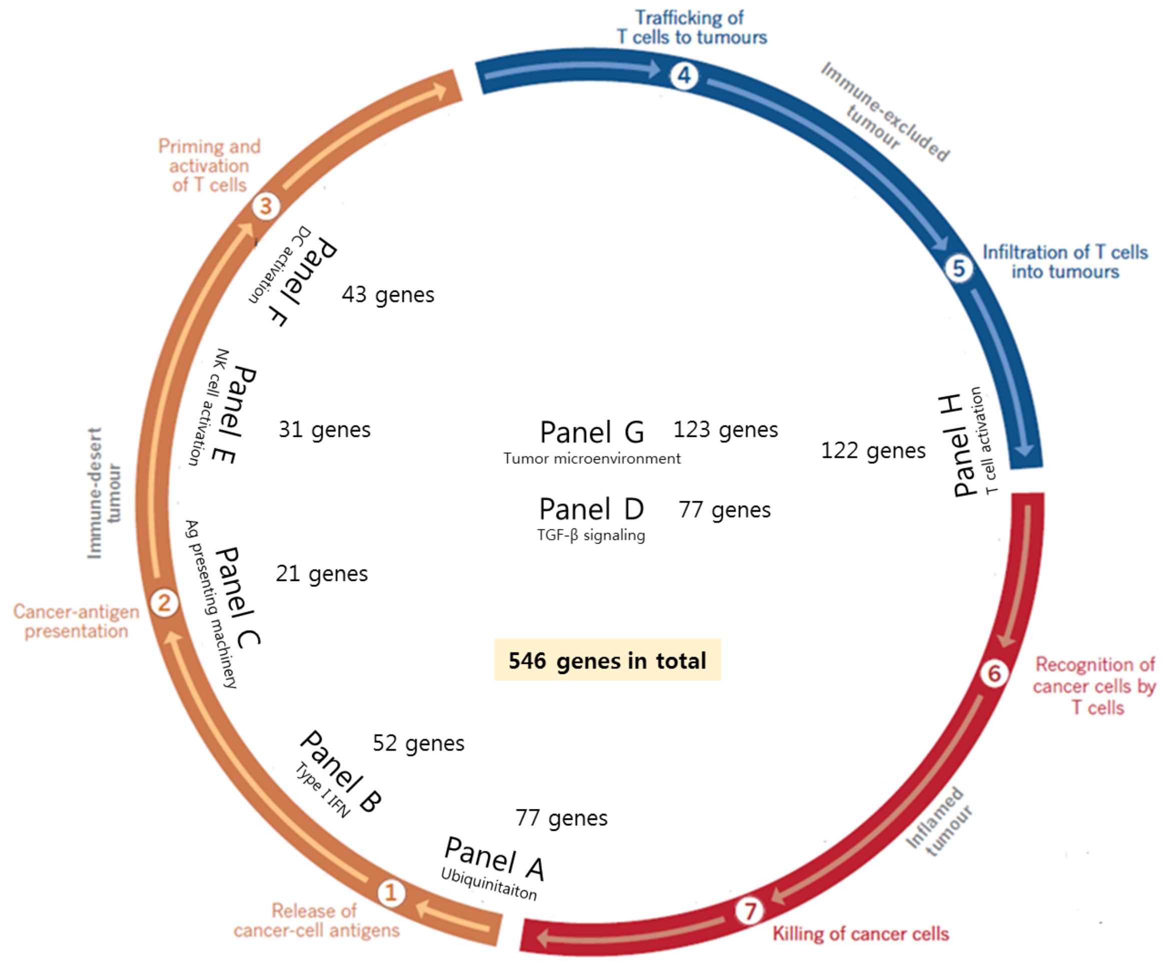

Figure S4. Three distinct subgroup-dependent cancer-immune gene expression within the individual panels of the cancer-immunity cycle in never smoker LUAD, Related to Figure 1

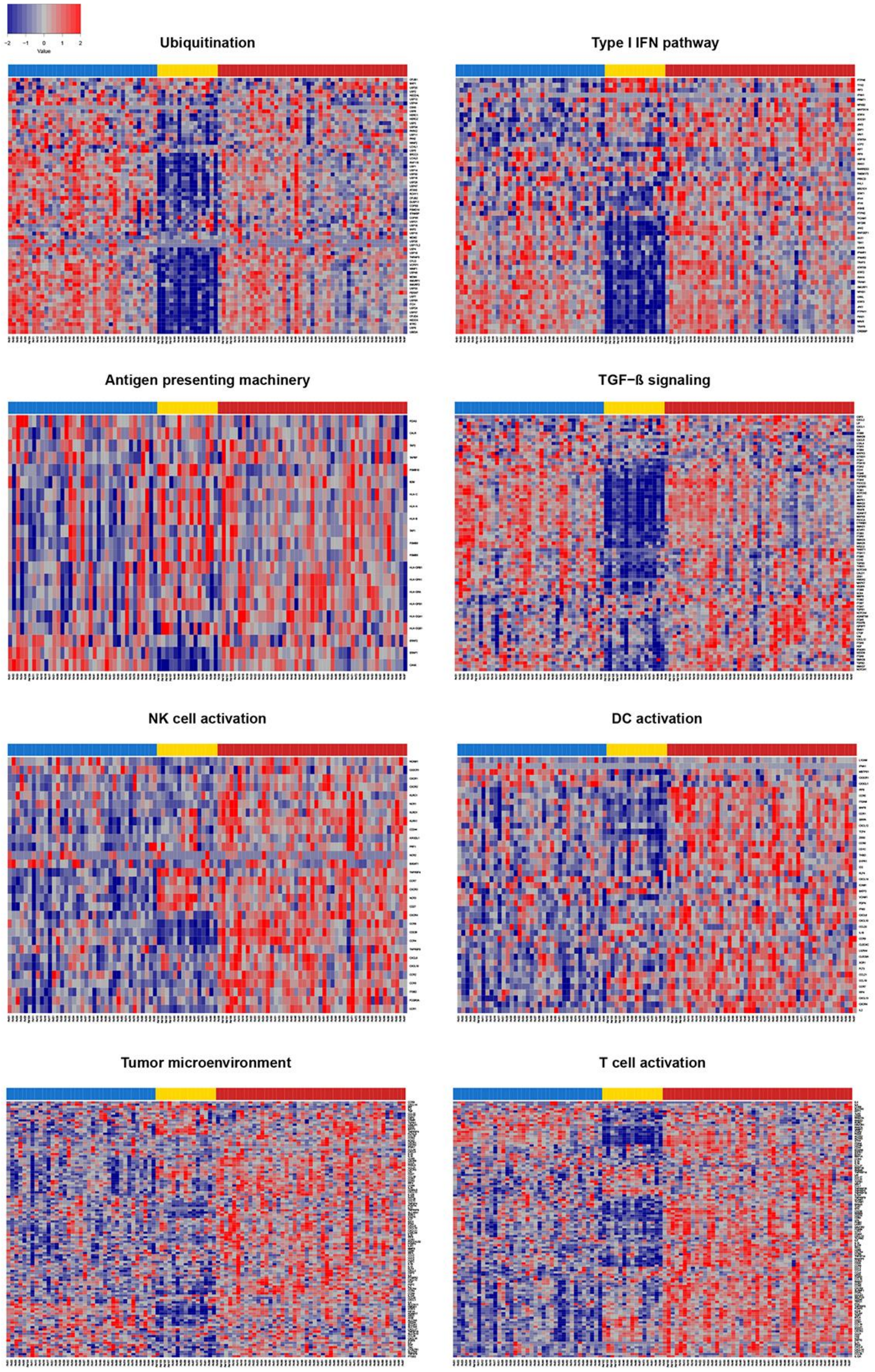

Table S1. Average number of mutations and neoantigens for each subgroup of patients, Related to Figure 2

	All	Subgroup		
		1	2	3
Mutations per patient	57.3	55.73	79.33	51.13
Neoantigens per patient	88.67	86.16	129.93	76.77
Neoantigens per mutated gene	3.65	3.57	3.66	3.72
Neoantigens per mutation	3.36	3.25	3.46	3.41
